# Comparative Assessment of Survival and Clinical Outcome Between Two Commercial Vitrification Kits with Different Warming Protocols After Blastocyst Culture: Potential Perspectives Toward Simplified Warming Procedures

**DOI:** 10.1007/s43032-023-01281-1

**Published:** 2023-06-13

**Authors:** Jan Gunst, Matthijs Vynck, Katleen Hostens, Valerie Standaert, Sylvie Roggeman, Arne van de Vijver

**Affiliations:** 1 Department of Laboratory Medicine, General Hospital Sint-Jan Brugge-Oostende, Ruddershove 10, 8000 Bruges, BE Belgium; 2 Department of Morphology, Imaging, Orthopedics, Rehabilitation and Nutrition, Faculty of Veterinary Medicine, Ghent University, Ghent, BE Belgium; 3 Department of Obstetrics and Gynecology – Center for Reproductive Medicine, General Hospital Sint-Jan Brugge-Oostende, Bruges, BE Belgium

**Keywords:** Blastocyst, Vitrification, Warming, Closed device, Live birth, Birth weight

## Abstract

This study investigates whether there is an effect on laboratory results and clinical outcome using commercial kits with similar vitrification but different warming procedures for blastocysts vitrified on day 5 or day 6. A single-center retrospective cohort study was performed between 2011 and 2020. A change from a stage-specific kit (Kit 1) to a universal kit (Kit 2) was undertaken in 2017. A total of 1845 untested blastocysts were warmed for single vitrified-warmed blastocyst transfers (SVBT). Eight hundred and twenty-five blastocysts were vitrified with Kit 1 and 1020 with Kit 2. Blastocyst survival was not different (96.1% versus 97.3%). Seven hundred seventy-seven SVBT were performed from Kit 1 and 981 from Kit 2. Overall clinical pregnancy and live birth rates were not different (35.4% versus 34.1% and 30.9% versus 30.5% for Kit 1 and 2, respectively). Subgroup analysis for live birth rates in relation to the day of blastocyst vitrification showed no differences (36.1% and 36.1% for day 5 and 25.4% and 23.5% for day 6 blastocysts, respectively). For both kits, the mean gestational age was not different (38.8 ± 2.5 weeks versus 38.8 ± 2.0 weeks) with a singleton birth weight of 3413 ± 571 g and 3410 ± 528 g for Kit 1 and Kit 2, respectively. Differences in warming procedures do not affect laboratory performance or clinical outcome after blastocyst vitrification. The plasticity of a human blastocyst may allow for further investigation on simplification of blastocyst warming procedures.

**Supplementary Information:**

The online version contains supplementary material available at 10.1007/s43032-023-01281-1.

## Introduction

Due to superovulation and a move toward single embryo transfer, cryopreservation of human embryos has become an established part of most IVF cycles [1, 2]. Vitrification at the blastocyst stage is considered the gold standard in human embryo cryopreservation [3]. Several kits with media for vitrification and warming are commercially available, which differ in several aspects from each other. In some kits, the composition of the vitrification solution (VS) is based on dimethylsulfoxide (DMSO) or glycerol (GLY) and ethylene glycol (EG), whereas others contain 1,2-propanediol (PROH) and EG. Warming solutions are usually based on sucrose or trehalose at decreasing concentrations. Exposure to cryoprotectant solutions is either performed at room temperature or at 37°C. For carrier devices, in the open system, the embryo can come in direct contact with liquid nitrogen, in contrast to a closed system where any contact to liquid nitrogen during vitrification, storage, and warming is strictly avoided.

To comply with national law regulating quality and safety requirements [4], the vitrification-warming process was validated in the laboratory prior to its use in clinical routine. A validation may involve minor changes or adaptations of the manufacturer’s protocols to obtain optimal performance in a given clinical environment. Furthermore, due to a possible long time-period during which cryopreserved human embryos are stored, it may be that blastocysts that were vitrified with one kit have to be warmed with another one with a potentially different saccharide concentration [5–7]. Therefore, validation should be done to ascertain the ability of the embryos to adapt to warming media with slight changes in composition as well as to minor changes in protocols. Also, because of legal necessity, a closed vitrification storage device was introduced. The validation is described of a blastocyst specific kit and a universal vitrification kit with a closed device, in order to have a safe and effective application. Further clinical results are reported until live birth including neonatal data of untested day 5 and day 6 vitrified blastocysts. The underlying mechanisms of these brand cryopreservation solutions and the impact of our findings are discussed in an attempt toward blastocyst warming protocol advancement.

## Methods

### Study Design

A retrospective monocentric cohort study was undertaken at the Center for Reproductive Medicine of the General Hospital Sint-Jan Brugge-Oostende, Bruges, Belgium, between January 2011 and December 2020 and included all patients that underwent vitrified-warmed blastocyst cycles with the aim to transfer a single blastocyst. All participating subjects gave written agreement for each fresh or frozen cycle. Exclusion criteria were culture to day 3 or day 7, double embryo transfer, pre-implantation genetic testing (PGT), re-vitrification, donor oocyte, oocytes from another clinic and post warming time to transfer exceeding 4.5 h. Repeat cycles of the same patient after a live birth were excluded. The study protocol was approved by the local Ethical Committee (Number 2565).

### Clinical Procedures

Patients were subjected to individualized ovarian stimulation schemes with urinary (Menopur®, Ferring Pharmaceuticals A/S, Copenhagen, Denmark) or recombinant FSH (Gonal F®, Merck-Serono, Geneva, Switzerland; Ovaleap®, Teva B.V., Haarlem, The Netherlands) in combination with GnRH antagonist (Cetrotide®, Merck-Serono, Geneva, Switzerland) or agonist (Suprefact®, Sanofi-Avantis, Frankfurt A/M, Germany; Gonapeptyl®, Ferring Pharmaceuticals A/S, Copenhagen, Denmark) for pituitary inhibition. When at least three follicles were ≥ 17 mm in diameter, ovulation was triggered by injection of hCG (Pregnyl®, Schering-Plough, Oss, The Netherlands) or Gonapeptyl in case of OHSS risk. Oocyte retrieval was performed 34–36 h after hCG by transvaginal ultrasound-guided ovum double lumen needle aspiration (Cook Medical, Ireland).

Recipients were subjected to substitute hormonal therapy. They were administered orally 3 times daily 2 mg of E2 valerate (Progynova®, Berlin, Bayer AG), starting on day 3. A transvaginal ultrasound was performed after one week. If an endometrial thickness of at least 7 mm was documented, vaginal progesterone supplementation (600 mg Utrogestan®, Besins Manufacturing, Belgium) was started to support the endometrial receptivity. Blastocyst transfer was performed on the 6^th^ day of progesterone supplementation. Progesterone and E2 valerate supplementation were continued for at least 14 days. If a positive ß-hCG could be detected, both hormonal supplementations were continued for another 12 weeks. For patients with a natural cycle a spontaneous LH surge was identified or hCG (Pregnyl; Ovitrelle®, Merck-Serono, London, United Kingdom) was administered. Blastocyst transfer was performed on the 6^th^ day after LH surge or the 7^th^ day after hCG administration.

### Blastocyst Culture and Transfer

All oocyte, embryo, and blastocyst handling outside the incubator was performed in holding medium (G-MOPS™ PLUS, Vitrolife, Sweden) under paraffin oil (OVOIL™, Vitrolife, Sweden) to maintain 37°C. Oocytes were identified and retrieved from the follicular aspirate. Overnight IVF was performed in fertilization medium (G-IVF™ PLUS, Vitrolife, Sweden). In case of ICSI, MII-oocytes were denuded using hyaluronidase (HYASE-10x™, Vitrolife, Sweden). Inseminated oocytes from IVF and ICSI and the derived embryos were individually cultured to the blastocyst stage in micro drops (G-1™ PLUS and G-2™ PLUS or G-TL™, Vitrolife, Sweden) under paraffin oil at 37°C in a 5.0% O_2_ and 6.5% CO_2_ non-humidified bench-top (G-185, K-Systems) or box-type incubator (HeraCell 240, Kendro). Depending on clinical demand, fresh embryo transfer was organized on day 3 or day 5 or postponed by a freeze all policy. For SVBT, a warmed blastocyst was cultured for 1 to 4.5 h in G-2 PLUS or G-TL. SVBT was performed using a guiding soft transfer catheter (Cook Medical, Ireland) and aspirating the embryo from a center-well dish in G-2 PLUS or G-TL under paraffin oil to maintain 37°C.

### Embryo Grading

Fertilization was confirmed based on the formation of two pronuclei and two polar bodies after 18 ± 2 h post insemination (hpi). Embryo development was assessed on day 2 at 44 ± 2 hpi and day 3 at 68 ± 2 hpi. From day 5 on, blastocysts were evaluated by a senior clinical embryologist according to a standard operating procedure based on the Gardner and Schoolcraft classification [8] following a liberal selection for day 5 or day 6 cryopreservation on registered vitrification times.

### Vitrification and Warming

#### Validation

The aim of the validation was to assess the vitrification-warming methodology with different ready-to-use kits. Kit 1 (RapidVit™ Blast/RapidWarm™ Blast, Vitrolife, Sweden) was validated in 2010, whereas Kit 2 (RapidVit™ Omni/RapidWarm™ Omni, Vitrolife, Sweden) was validated in 2016. Hereby, the compatibility of vitrification Kit 1 with warming Kit 2 was also examined. A Rapid-i™ (Vitrolife, Sweden) was used as carrier and storage device. Material for validation was derived from patients who consented for research in case of inappropriate fresh or by law expired frozen embryos after a 5-year storage. Results of both validations were compared. The primary outcome of the validation was immediate morphological survival (survival rate) after warming based on membrane intactness without extensive cellular degeneration. Secondary outcomes included the functional survival parameters, i.e., re-expansion and, if available, 24-h development.

Acceptance criteria for morphological survival during validation were 70% for competence level and 95% for benchmark, as suggested by the Alpha consensus meeting [9]. Additionally, the minimum criteria for re-expansion (2–4 h) and development until 24 h post warming were set at 70% [10] and 80% [11], respectively.

#### Vitrification

To optimize survival rates, laser blastocyst collapsing (Saturn, Research Instruments, UK) was performed prior to vitrification on blastocysts with expansion. Blastocysts were subjected to a single laser pulse aimed toward a junction between TE cells. Without expansion, vitrification was started immediately.

The microdroplet vitrification method under oil at 37°C [12] was developed first for vitrification Kit 1 followed by vitrification Kit 2, preparing 25 μl single-use microdroplets of solutions 1 and 2 for up to 6 blastocysts, covered by paraffin oil. For solution 3, two single-use droplets without oil, 200 μl to rinse off the oil from the pipette and a 20 μl dehydration droplet were made in an ICSI dish just before use, from a tube with solution 3 kept at 37°C. Prior to moving blastocysts from one solution to another, a glass pipette was primed with the next solution. Before each new vitrification, the pipette was rinsed in the previous solutions 1 and 2. Exposure times during vitrification procedures for both kits involved the recommended exposure times by the manufacturer of respectively: 5–20 min, 2 min, and 45 s. For vitrification, blastocysts were loaded on a Rapid-i carrier in a 30 nL droplet [13]. Subsequent vitrification and sealing were carried out according to the Rapid-i instructions.

#### Warming

The microdroplet warming technique was developed initially for vitrification Kit 1 followed by vitrification Kit 2, starting in a 200 μl open air single-use droplet of solution 1 in a dish. The first step was followed by using 25 μl single-use microdroplets of solutions 2, 3, and if applicable 4, covered by paraffin oil to maintain 37°C. With Kit 1, the recommended exposure times by the manufacturer were used, respectively: 2, 3, and 5 min, whereas for Kit 2, a validated amended time schedule was used, i.e., 1, 2, 3, and 5 min.

Warming started by opening the straw seal with a veterinary cutter of the Rapid-i straw standing in liquid nitrogen close to the microscope. The Rapid-i carrier was lifted with the noose pincer to fix in one hand. Using both hands, the Rapid-i straw and carrier were together removed from the liquid nitrogen, immediately brought to the warming dish, and then, the carrier was fluidly removed from the outer straw and dipped into the 200 μl open air warming solution. The whole procedure is performed within one second. Next, the blastocyst was moved to solutions 2, 3, and 4 according to the aforementioned time steps.

All dishes were prepared and pre-heated in a mini-incubator (G-85, K-Systems) under atmospheric conditions. Test tubes containing vitrification solution 3 and warming solution 1 were warmed in a block heater (BT3, Grant).

### Clinical and Neonatal Outcome

The primary outcome was live birth, defined as the birth of a newborn. Secondary outcomes analyzed were as follows: +ß-hCG pregnancy (between 5.8 and 71.2 U/l, 13–16 days after SVBT); implantation (number of gestational sacs at 7 weeks); and clinical pregnancy (presence of a gestational sac with fetal heartbeat). Clinical miscarriage was defined as spontaneous and induced abortion after positive heartbeat had been detected.

Neonatal data were recorded for gestational age (GA), birth weight (BW), sex, and complications during pregnancy. Gestational age was determined by subtraction of the date of cycle day 1 from the date of birth. The sex ratio was defined as the ratio of females to males. Induced abortions and congenital malformations at birth were registered as complications.

### Statistical Analysis

For categorical data in 2×*n* (*n* ≥ 2) table format, differences between kits were assessed using Pearson’s chi-squared tests with continuity correction. Fisher’s exact test was used when table cells with less than 10 observations were present. Live birth rate was modeled using multivariate logistic regression adjusting for potential confounding factors. Potential confounding factors considered were the day of vitrification, maternal age, and the vitrification-warming kit. Odds ratios and their profile likelihood confidence intervals were obtained from the logistic regression model. Differences in neonatal outcomes (mean gestational age, mean birth weight) between day 5 and day 6 were compared using Welch two-sample *t*-tests. For all tests, a *p*-value < 0.05 was considered statistically significant. Where applicable, all tests were conducted using a two-sided alternative hypothesis. All analyses were performed in R version 4.0.3.

## Results

The single-use microdroplet method was initially validated when changing from slow freezing- thawing to vitrification-warming using Kit 1. When moving to vitrification-warming using Kit 2, the use of the single-use microdroplet protocol for Kit 2 was validated against Kit 1 (Table 1). Morphological survival rates for all validations were above the 95% benchmark and no difference in re-expansion was observed. Also, the combination of vitrification Kit 1 with warming Kit 2 was approved for morphological survival (95.8%) and for 24h development (83.3%). In clinical routine, this cross-over group comprised 122 SVBT (excluding one survived blastocyst without inner cell mass and trophectoderm cell cohesion, i.e. structural loss at transfer), which were included in the Vitrification Kit 1 group for the general evaluation. A sub-evaluation of the cross-over group is shown in Supplementary Table 1. No significant differences in +ß-hCG pregnancies (50.8%, *p*=0.72), clinical pregnancies (29.5%, *p*=0.17), and live birth rates (26.2%, *p*=0.27) were observed.

**Table 1:** Validation data for Kit 1 and Kit 2

	Vitrification Kit 1 Warming Kit 1	Vitrification Kit 2 Warming Kit 2	Vitrification Kit 1 Warming Kit 2	*p*-value*
Embryos	40	40	51	
Recovered (%)	37/40 (92.5)	40/40 (100.0)	48/51 (94.1)	0.24
Survival (%)	37/37 (100.0)	38/40 (95.0)	46/48 (95.8)	0.41
Re-expansion (%)	33/37 (89.2)	35/40 (87.5)	38/48 (79.2)	0.37
24-h development (%)	-	-	40/48 (83.3)	

*p-value determined by means of Pearson’s chi-squared test

Characteristics of the underlying fresh cycles for those patients that received a SVBT with Kit 1 or Kit 2 in clinical routine are shown in Table 2. Between January 2011 and December 2017, 460 patients underwent 825 vitrified-warmed Kit 1 blastocyst cycles. While afterwards until December 2020, 1020 vitrified-warmed Kit 2 blastocyst cycles were performed for 488 patients. Maternal age in the Kit 1 group (31.2 ± 4.5 y) and in the Kit 2 group (31.8 ± 4.8 y) differed significantly (*p*=0.024). The culture time at day 5 and day 6 for the Kit 1 group (116.9 ± 1.6 h; 140.6 ± 1.3 h) and Kit 2 group (116.8 ± 1.2 h; 140.5 ± 1.1 h) were similar (*p*=0.38 and *p*=0.61). Finally, the post warming time for Kit 1 (2.3 ± 0.7 h) compared with Kit 2 (2.4 ± 0.4 h) again differed significantly (*p*=0.012). However, the small but significant difference for both young patient age populations and post warming time between the Kit 1 and Kit 2 group have no biological relevance. Multivariate logistic regression analysis, shown in Table 3, revealed that maternal age (*p* < 0.001) and days of culture (*p* < 0.001) were main factors affecting LBR after SVBT, but not the kit used (*p*=0.99).

**Table 2:** Characteristics of patients and the fresh cycles using Kit 1 or Kit 2

	Vitrification Kit 1 Warming Kit 1/Kit 2	Vitrification Kit 2 Warming Kit 2	*p*-value*
Number of patients	460	488	
Maternal age (year)			
Mean ± SD	31.2 ± 4.5	31.8 ± 4.8	0.024
Time of vitrification (h)			
Day 5 mean ± SD	116.9 ± 1.6	116.8 ± 1.2	0.38
Day 6 mean ± SD	140.6 ± 1.3	140.5 ± 1.1	0.61
Post warm time (h)			
Mean ± SD	2.3 ± 0.7	2.4 ± 0.4	0.012

*p-value determined by means of Welch two-sample t-test

**Table 3:** Multivariate logistic regression analysis of cycle characteristics

Confounder	Odds ratio estimate	95% confidence interval	*p*-value*
Maternal age	0.46	[0.34 to 0.62]	< 0.001
Kit 1/Kit 2	1.00	[0.81 to 1.23]	0.99
Days of culture	0.57	[0.46 to 0.70]	< 0.001

*p-value determined by means of logistic regression

Table 4 shows the main results and clinical outcome with no difference in rates of survival (96.1% and 97.3%, *p*=0.23), and similar rates of +ß-hCG pregnancies (48.9% and 47.4%, *p*=0.56), clinical pregnancies (35.4% and 34.1%, *p*=0.62), miscarriages (12.4% and 8.7%, *p*=0.17), implantation (36.6% and 35.2%, *p*=0.51), and live birth (30.9% and 30.5%, *p*=0.89) respectively, for the Kit 1 and Kit 2 group.

**Table 4:** Main results and clinical outcome for vitrification/warming using the different kits

	Vitrification Kit 1 Warming Kit 1/Kit 2°	Vitrification Kit 2 Warming Kit 2	*p*-value*
Warmed blastocysts	825	1020	
Recovered blastocysts (%)	820/825 (99.4)	1011/1020 (99.1)	0.23
Survived blastocysts (%)	788/820 (96.1)	984/1011 (97.3)	0.23
Transferred blastocysts (%)	777/820 (94.8)	981/1011 (97.0)	0.02
+ßhCG pregnancies (%)	380/777 (48.9)	465/981 (47.4)	0.56
@ Day 5 (%)	211/399 (52.9)	289/543 (53.2)	0.97
@ Day 6 (%)	169/378 (44.7)	176/438 (40.2)	0.22
Implantation (%)	284/777 (36.6)	345/981 (35.2)	0.51
Clinical pregnancies (%)	275/777 (35.4)	335/981 (34.1)	0.62
@ Day 5 (%)	163/399 (40.9)	217/543 (40.0)	0.84
@ Day 6 (%)	112/378 (29.6)	118/438 (26.9)	0.44
Miscarriage rate (%)	34/275 (12.4)	29/335 (8.7)	0.17
*@ Day 5 (%)*	18/163 (11.0)	15/217 (6.9)	0.22
*@ Day 6 (%)*	16/112 (14.3)	14/118 (11.9)	0.73
Live births (%)	240/777 (30.9)	299/981 (30.5)	0.89
@ Day 5 (%)	144/399 (36.1)	196/543 (36.1)	1.00
@ Day 6 (%)	96/378 (25.4)	103/438 (23.4)	0.59
Singleton births (%)	233/240 (97.1)	291/299 (97.6)	1.00
Multiple gestation (%)	7/240 (2.9)	8/299 (2.7)	1.00
Male to female ratio	133/114 (1.17)	139/168 (0.83)	0.05

°702 blastocysts were warmed with Warming Kit 1 and 123 with Warming Kit 2

*p-value determined by means of Pearson’s chi-squared test

There are also no differences in neonatal outcomes between Kit 1 and Kit 2 (Table 5), for gestational age (38.8 w ± 2.3 w and 38.8 w ± 2.0 w, *p*=0.95), singleton birth weight (3413 g ± 571 g and 3410 g ± 528 g, *p*=0.95), and malformations (1.7% and 0.7%, *p*=0.42). Details regarding malformations and the reason for induced abortions are shown in Supplementary Table 2.

**Table 5:** Neonatal outcome after vitrification/warming using the different kits

	Vitrification Kit 1 Warming Kit 1/Kit 2°	Vitrification Kit 2 Warming Kit 2	*p*-value
Gestational age (mean ± SD)	38.8 ± 2.3 weeks	38.8 ± 2.0 weeks	0.95*
Birth weight ALL	3349 ± 639g	3350 ± 579g	0.98*
	1 unknown		
Singleton birth weight	3413 ± 571g	3410 ± 528g	0.95*
@ Day 5	3419 ± 511g	3381 ± 515g	0.51*
@ Day 6	3404 ± 657g	3464 ± 551g	0.50*
	1 unknown		
Malformation (%)	4/240 (1.7)	2/299 (0.7)	0.42^#^
Induced abortions (%)	3/240 (1.3)	2/299 (0.7)	0.66^#^

°702 blastocysts were warmed with Warming Kit 1 and 123 with Warming Kit 2.

*p-value determined by means of Welch two-sample t-test

^#^*p*-value determined by means of Pearson’s chi-squared test

Remark: detailed information on the ID and type of malformations/cause of induced abortion are shown in Supplementary Table 2

Subgroup analysis for day 5 and day 6 blastocysts is in favor of day 5 but confirm no differences between the Kit 1 and Kit 2 group for clinical outcome and neonatal data: +ß-hCG pregnancies (52.9% and 53.2%, *p*=0.97), clinical pregnancies (40.9% and 40.0%, *p*=0.84), miscarriage rate (11.0% and 6.9%, *p*=0.22), live birth rate (36.1% and 36.1%, *p*=1.00), and singleton birth weight (3419 ± 511g and 3381 ± 515g, *p*=0.51).

## Discussion

In the field of human IVF, vitrification/warming has become the first choice for cryopreservation of oocytes, cleavage stage embryos, and blastocysts, and in many aspects, it is considered to be more successful than the conventional slow freezing/thawing procedure [14]. Today, there are several commercial kits available with variations regarding protocol, cryoprotectant agent (CPA) type and concentrations, exposure temperature and time, and different (open/closed) carriers, which may all affect the efficacy of a vitrification/warming program. Particularly in Europe, the use of approved, CE-marked products is mandated for use in human reproduction by national law, implementing Europeans Tissues and Cells Directive 2004/23/EC on quality and safety [15]. This also applies for vitrification and warming media and storage devices. Kits may differ in one way or another and due to the long-time of cryostorage, vitrification/warming kits may change or even be no longer available.

In the current study, we compared the use of a blastocyst-specific vitrification-warming kit against a universal vitrification-warming media kit regarding recovery and survival rates, as well as clinical outcomes after SVBT. In line with the most widely used 1.5M PROH–0.1M sucrose protocol for slow freezing [16], our preference went toward vitrification kits composed of equal and increasing concentrations of the permeable CPAs PROH and EG. Recently, this DMSO-free CPA combination has been found [17] to have the lowest ice crystal growth velocity and thus decreased ice crystal propagation compared to vitrification solutions containing DMSO either alone or in combination with other CPAs.

Both kits are designed for use at 37°C throughout the whole procedure. Exposure of cells to permeating CPA at higher temperature increases permeation, which reduces exposure timing and results in more physiologic conditions. Using this methodology, exposure time to cryoprotectant solutions during vitrification procedures is limited to 2 min and 45 s for both kits during equilibration and vitrification respectively. This high procedural time efficiency can lead to less distraction for the embryologist [18].

For each kit, the working conditions in the laboratory must be adapted in order to achieve good results. To optimize these conditions and sustain an environment of 37°C, we developed a microdroplet vitrification/warming method using an oil overlay, which helps to control and maintain osmotic and temperature stability during dehydration and rehydration [19]. For practical reasons, the oil overlay is skipped for the final vitrification and the first warming solution. We considered the introduction of the single-use microdroplet setting under oil crucial to maintain stable conditions and to achieve standardized results across all embryologists. Another advantage of this technique is the standardization of the operating procedure for each embryo by using single-use droplets allowing a guaranteed traceability per embryo.

As fixed exposure times are recommended, a complete spontaneous collapse per blastocyst cannot be assured. In this study, we applied laser-assisted artificial blastocyst collapse prior to vitrification following reports of suboptimal survival without collapse [20–23]. Furthermore, blastocyst collapse was recently recommended for low-molarity VS (<6.5M), estimated around 5.1M, in order to get a better penetration of CPA into the cellular compartments [24]. The benefit of blastocyst collapse has been confirmed in a recent meta-analysis [25].

Differences in the chances to obtain a pregnancy when transferring embryos vitrified on day 5 or day 6 have been shown before [26–28] and in some meta-analysis [29, 30]. Our results confirm these observations. On the other hand, once an ongoing pregnancy is established, development to term and birth weight is not different from blastocysts vitrified on day 5.

Overall, health of children born after cryopreservation is reassuring [31]. In Belgium, collection of data on pregnancy and neonatal outcome after ART is mandatory. The results from our study are in line with other published data and demonstrate there is no effect of different warming procedures including exposure to different sucrose concentrations on the chance of implantation, development to term or neonatal outcome.

Sex ratio of children born was almost significantly different between Kits 1 and 2. The debate on the effect of blastocyst transfer on sex ratio is ongoing [32, 33]. Sex of the embryos is determined at the time of fertilization. With no changes in selection criteria of embryos for vitrification and similar results after warming, the vitrification and warming procedures are not contributing to differences in sex ratio and are therefore considered a chance finding.

Despite these promising and comparable results for both kits, we need to be aware of the retrospective nature of this study. Cycles after a live birth from the same patient were excluded from the analysis as such patients more likely can have another live birth compared to other patients. Inclusion of such cycles could result in an overestimated expected live birth rate at the patient level. Of note, live birth rates with and without the inclusion of these cycles were on par in our cohort (data not shown). The total blastocyst population was analyzed for cryotolerance on day 5 and day 6. The majority of embryos included expanding blastocysts (data not shown). The hydrodynamic behavior and cryotolerance of different developmental blastocyst stages still have to be assessed.

Another limitation can be the lack of detail of the commercial product composition. Information on components can be available on the certificate of analysis of respective products and concentrations of certain components are sometimes published. For vitrification, levels of cryoprotectants in different commercial kits are usually in the same range. The major difference between Kits 1 and 2 is in the warming solutions. Kit 1 was developed for blastocysts, and the composition based on work by Lane et al. [34] where warming solutions contain a sucrose level starting with 0.25 M [34–36]. Kit 2 was developed for all stages from oocytes to blastocysts. For oocytes, typically a starting concentration of 1M sucrose is used for warming [37] and this concentration has also proven successful for blastocysts [38] or other stages such as 2-PN-oocytes [39], cleavage-stage [40], or morula stage embryos [41]. Warming media of both kits have decreasing sucrose concentrations and differ in the number of steps: 0.25M-0.125M-0M in 3 steps for the blastocyst-specific Kit 1 and 1M-0.5M-0.25M-0M in 4 steps for the universal Kit 2.

Commonly, sucrose has been accepted as a highly effective non-permeating saccharide in removing intracellular CPAs (PROH, DMSO, EG, GLY) and enabling stepwise rehydration to support isotonic equilibration. The 4-step protocol with decreasing sucrose concentrations has been accepted as a universal warming/dilution media system for human cleavage-stage embryos and blastocysts [6, 42, 43]. Parmegiani and colleagues already showed in 2014, that even oocytes that were cryopreserved by conventional slow freezing could be successfully thawed with a modified 4-step protocol with warming solutions initially designed for warming of vitrified oocytes [4]. Oocytes are more vulnerable to osmotic stress and intracellular ice crystal formation, which explains the need for more specific warming solutions. However, the same solutions can be used for human blastocysts, whose lipid membranes show a different morphological behavior. This can explain the similar survival rates that we observed in our study comparing the blastocyst kit and the universal kit despite different steps and sucrose gradient concentrations. It may also explain why different commercial warming kits are interchangeable for human blastocysts [42]. Additionally, our results indicate that blastocysts tolerate warming solutions with different sucrose levels as well as different working temperatures. This tolerance is direct proof for the adaptable ability of human embryos and without such an adaptability, the cryo-survival, clinical pregnancy, and live birth rate would have been affected. The experimental data of Jin and Mazur in 2015 on mouse oocytes and embryos were the first to prove that osmotic intracellular dehydration prior to cooling together with the rate of warming were essential factors for success [44]. In agreement with these findings, we confirm that the same type and concentration of permeating CPA in Kit 1 and Kit 2 could be possible cooperating factors in prior mentioned suboptimal conditions. Furthermore, clinical studies on blastocyst vitrification and warming already showed the independence of permeating CPA composition with low or high osmolarities of non-permeating CPA [35, 36, 38, 41, 45]. The increased CPA exchange with increased working temperature together with the plasticity of blastocyst cells could allow potential perspectives toward a more rapid and simplified method without saccharide gradients for blastocyst warming. Recently, this has been demonstrated preclinically [46–49] and clinically by Manns [50].

In conclusion, the use of different kits for vitrification and warming of blastocysts resulted in similar cryobiological as well as clinical outcome of babies born, which underlines the adaptable ability of human embryos. Validation and use of a microdroplet method support standardization of the method, minimizing variation between operators while providing stable laboratory conditions and results. Use of different warming solutions and exposure times results in similar clinical outcome with no effect on birth weight or health of children born. Our observations, combined with recent findings on simplified warming procedures, demonstrate that warming of blastocysts is possible under different conditions and opens possibilities for further optimization of laboratory procedures.

### Supplementary information

ESM 1 (DOCX 16 kb)

## Data Availability

All data generated or analyzed during this study are included in this published article (and its supplementary information files).
