# Supplementary material for: Comparative Assessment of Survival and Clinical Outcome Between Two Commercial Vitrification Kits with Different Warming Protocols After Blastocyst Culture: Potential Perspectives Toward Simplified Warming Procedures
Source: Reprod Sci. 2023 Jun 13;30(11):3212–21. doi: 10.1007/s43032-023-01281-1 (PMC10643310; doi:10.1007/s43032-023-01281-1)
Supplement: Supplementary file 1 — (DOCX 16 kb) [file 43032_2023_1281_MOESM1_ESM.docx]

**Supplementary Table 1: Results after vitrification with Kit 1 and warming with either Kit 1 or Kit 2**

|  | Vitrification Kit 1  Warming Kit 1 | Vitrification Kit 1  Warming Kit 2 | *p*-value* |
| --- | --- | --- | --- |
| Warmed blastocysts | 702 | 123 |  |
| Recovered blastocysts (%) | 697/702 (99.3) | 123/123 (100.0) | 1 |
| Survived blastocysts (%) | 665/697 (95.4) | 123/123 (100.0) | 0.061 |
| Transferred blastocysts (%) | 655/697(94.0) | 122/123 (99.2) | 0.014 |
| +ßhCG pregnancies (%) | 318/655 (48.5) | 62/122 (50.8) | 0.72 |
| @ Day 5 (%) | 177/336 (52.7) | 34/63 (54.0) | 0.96 |
| @ Day 6 (%) | 141/319 (44.2) | 28/59 (47.4) | 0.75 |
| Implantation (%) | 249/655 (38.0) | 37/122 (30.3) | 0.13 |
| Clinical pregnancies (%) | 239/655 (36.5) | 36/122 (29.5) | 0.17 |
| @ Day 5 (%) | 143/336 (42.6) | 20/63 (31.7) | 0.14 |
| @ Day 6 (%) | 96/319 (30.1) | 16/59 (27.1) | 0.76 |
| Miscarriage rate (%) | 30/239 (12.6) | 5/37 (13.5) | 0.79 |
| @ Day 5 (%) | 17/143 (11.9) | 1/20 (5.0) | 0.70 |
| @ Day 6 (%) | 13/96 (13.5) | 4/17 (23.5) | 0.28 |
| Live births (%) | 208/655 (31.8) | 32/122 (26.2) | 0.27 |
| @ Day 5 (%) | 125/336 (37.2) | 19/63 (30.1) | 0.35 |
| @ Day 6 (%) | 83/319 (26.0) | 13/59 (22.0) | 0.63 |
| Multiple gestation (%) | 6/208 (2.9) | 1/32 (3.1) | 1 |

*p-value determined by means of Pearson’s chi-squared test

**Supplementary Table 2: Details on ID / type of malformations and cause of induced abortions**

| Vitrification Kit 1  Warming Kit 1 / 2 | Vitrification Kit 2  Warming Kit 2 |
| --- | --- |
| Malformation ID and type | |
| [745.4] Ventricular Septal Defect | [745.1] Transposition of great vessels |
| [745.4] -[745.5] Ventricular Septal Defect - Ostium Secundum Type Atrial Septal Defect | [747.2] Other Anomalies Of Aorta |
| [753.2] Obstructive Defects Of Renal Pelvis and Ureter |  |
| [755.1] Syndactyly |  |
| ID and type of cause for induced abortion | |
| [758.0] Trisomy 21 | [758.2] Trisomy 18 |
| [xxxx] Cerebral malformation | [758.2] Trisomy 18 |
| [xxxx] Mega bladder, heart defect  [755.01] polydactyly  [756.79] omphalocele |  |
